# An update of the Japanese Oslo Sports Trauma Research Center questionnaires on overuse injuries and health problems

**DOI:** 10.1371/journal.pone.0249685

**Published:** 2021-04-01

**Authors:** Sonoko Mashimo, Naruto Yoshida, Takaaki Hogan, Ayaka Takegami, Satoru Nishida, Yasuharu Nagano

**Affiliations:** 1 Institute for Liberal Arts and Sciences, Osaka Electro-Communication University, Neyagawa, Osaka, Japan; 2 Faculty of Health Care, Teikyo Heisei University, Toshima, Tokyo, Japan; 3 Media Communication Center, Osaka Electro-Communication University, Neyagawa, Osaka, Japan; 4 Graduate School of Comprehensive Human Sciences, University of Tsukuba, Tsukuba, Ibaraki, Japan; 5 Faculty of Sports and Health Science, Fukuoka University, Fukuoka, Fukuoka, Japan; 6 Department of Sports and Health Science, Japan Women’s College of Physical Education, Setagaya, Tokyo, Japan; National Cheng Kung University College of Medicine, TAIWAN

## Abstract

Monitoring the health of athletes is important for their protection, and questionnaires such as those produced by the Oslo Sports Trauma Research Center (OSTRC) are a valuable tool in this process. In 2020, several changes were made to the OSTRC questionnaires (OSTRC-O, OSTRC-H), including changes to the wording, structure, and logic of the original questionnaires. In the present study, the Japanese versions of the OSTRC questionnaires (OSTRC-O.JP, OSTRC-H.JP) were revised to meet the requirements of the updated versions and to analyse new and previously collected data to illustrate the impact of the changes on Japanese athletes. Proposed changes were categorized as minor or more substantial; minor changes were effected to the questionnaire instructions and to the wording of all four questions, and more substantial changes were made to the wording of question 2. The updated questionnaires also included changes to questionnaire logic and answer categories. To assess the consequences of the changes to the wording of question 2, 101 athletes were asked to complete the OSTRC-H.JP, which included both the original and updated versions of question 2, over 10 consecutive weeks. We calculated the number of health problems identified when new gatekeeper logic was and was not applied, using 1585 OSTRC-H.JP responses to assess the consequences of the changes to the questionnaire logic. The kappa coefficient, which measures the level of agreement between the responses to question 2 of the original and updated versions, was high. By applying gatekeeper logic, there was a remarkable reduction in the number of injuries and illnesses among all health problems but less reduction in substantial health problems and time loss health problems. These changes will make it easier for Japanese athletes to complete the questionnaires and improve the quality of collected data.

## Introduction

Epidemiological studies for injury and illness in sports are critical elements to protect the health of athletes [1, 2]. The methods for investigating the extent of injury and illness have been published in consensus statements for several sports including football [3], rugby union [4], and golf [5] and also in those from the International Olympic Committee as a method of investigating multisport events [2, 6]. There are three consensus-recommended injury and illness definitions: all physical complaints regardless of their consequences (*any complaint* definition), injuries or illnesses leading to the athlete seeking attention from a qualified medical practitioner (*medical attention* definition), and injuries or illnesses leading to the athlete being unable to complete the current or future training session or competition (*time-loss* definition) [2, 7]. Traditionally, injury surveillance systems commonly rely on the definition of ‘time-loss’, the narrowest of all consensus-recommended definitions [8–10]. However, traditional injury surveillance systems lack the ability to estimate the full impact of overuse injuries because athletes with overuse injuries can often continue to participate in their sports despite the existence of such problems [3, 11, 12].

To address these challenges, the Oslo Sports Trauma Research Center (OSTRC) Overuse Injury Questionnaire (OSTRC-O) was developed and validated by Clarsen *et al*. [7] in 2013 to record the magnitude of overuse injuries. The method was to periodically administer the questionnaire to an entire group of participants throughout the surveillance period and employed a broad definition of injury to record all physical complaints in predefined anatomical areas. Distributing the questionnaire regularly (e.g. weekly) allows clinicians and researchers to monitor how the consequences of overuse injury change over time [12]. The Oslo Sports Trauma Research Center Questionnaire on Health Problems (OSTRC-H) was also developed in 2014 to record all types of health problems (injury and illness) [13]. The effectiveness of this questionnaire was prospectively evaluated by Clarsen *et al*. [13] with their Norwegian Olympic and Paralympic athletes preparing for the 2012 London Games. The OSTRC questionnaires (OSTRC-O and OSTRC-H) have been translated into several languages and been widely adopted in sports injury research [12, 14–18].

In 2020, several changes were made to the OSTRC questionnaires, including changes to their wording, structure, and logic [12]. The OSTRC questionnaires have become widely used in both research and clinical environments, and this has led to the need for their improvement, ranging from clarity and consistency of wording to principles of data analysis [12]. The updated versions of the questionnaires were named OSTRC-O2 and OSTRC-H2, respectively. The original English versions of the OSTRC questionnaires had been previously translated and culturally adapted into the Japanese context [17, 18]. To use the updated versions of the OSTRC questionnaires among Japanese athletes, it is necessary to translate them into Japanese and guarantee the content, conceptual, and semantic equivalence with the updated English versions. Therefore, the Japanese versions of the OSTRC questionnaires (OSTRC-O.JP and OSTRC-H.JP) were revised in this study to meet the requirements of the updated versions, and newly and previously collected data were analysed to identify the impact of the changes on Japanese athletes.

## Materials and methods

### Update of the OSTRC questionnaires

The OSTRC-O was developed to capture the extent of overuse injuries in sports [7]. It consists of four questions that seek to evaluate the consequences of overuse injuries: (1) sports participation, (2) training volume, (3) sports performance, and (4) pain [7, 12]. A severity score is calculated from 0 to 100 based on these four questions. The OSTRC-H was developed by modifying the OSTRC-O to record all types of injuries and illnesses [13]. This questionnaire also consists of four questions, and if an athlete reports a health problem, then he/she is required to provide additional information such as the type of problem and its location or main symptoms.

The updated versions of the questionnaires are OSTRC-O2 and OSTRC-H2, respectively. Proposed changes were categorized as minor or more substantial [12]. Minor changes were made to the questionnaire instructions and to the wording of all four questions to correct the ambiguity and inconsistency between questions in the original questionnaires [12]. More substantial changes were made to the wording of question 2; instead of asking about the extent to which athletes have reduced their training volume, the revised question asks about the extent to which athletes have modified their training or competition [12].

The updated questionnaires also include changes to questionnaire logic and answer categories [12]. As questions 2–4 are only relevant for athletes who have a health problem and continue to participate in training and competition, a new gatekeeper logic that can be applied to question 1 was proposed. If an athlete selects the first answer option ‘full participation without health problems’ in question 1, all further questions are redundant. In this case, a total severity score of 0 is assigned and the questionnaire is complete. If an athlete selects the fourth answer option ‘could not participate due to a health problem’ for question 1, questions 2–4 are redundant. In this case, a total severity score of 100 is assigned. The athlete then continues directly to the additional questions researchers may apply to the questionnaire to classify the reported health problem [12]. For consistency and clarity, the response category ‘cannot participate at all’ was removed from questions 2 and 3. Accordingly, the values to calculate severity score were aligned with questions 1 and 4 (i.e. a = 0, b = 8, c = 17, d = 25) [12].

### Athletes and recruitment

We approached the team coaches or athletic trainers from two university basketball teams and one university football team and asked whether they were interested in participating in the study. After they expressed interest, an introductory meeting was held for each team with the coaches or athletic trainers and the athletes to present the purpose of the study and ask their consent to participate. The inclusion criteria were as follows: age over 18 years, competing at sub-elite or elite level, and ability to speak and understand the Japanese language [14, 18]. Athletes were included regardless of whether they had present or previous injuries. The participants’ demographics are summarized in Table 1.

**Table 1:** Participants’ characteristics.

Sex (*n*)	Male 86
	Female 15
Basketball (*n*)	44
Football (*n*)	57
Age, years (mean±SD)	19.1 ± 1.1
Height, m (mean±SD)	1.73 ± 0.08
Weight, kg (mean±SD)	67.6 ± 9.3
Sports experience, years (mean±SD)	11.3 ± 2.5
Training volume, hours/week (mean±SD)	12.4 ± 4.2

SD, standard deviation.

### Ethical approval

This study was approved by the Ethics Committee of Osaka Electro-Communication University (approval number: 20–001). All participants provided written informed consent before participating in the study. The study was conducted in accordance with the principles outlined in the Declaration of Helsinki.

### More substantial changes

For question 2, in which more substantial changes were made, the English versions of OSTRC questionnaires (OSTRC-O2, OSTRC-H2) [12] were used for Japanese translation. The translation process was conducted according to the guidelines presented by Beaton *et al*. [19, 20] and the principles of good practice laid down by the International Society for Pharmacoeconomics and Outcome Research [21]. The final updated questionnaires of the Japanese versions (OSTRC-O2.JP, OSTRC-H2.JP) are shown in S1 and S2 Files.

The participants were asked to complete the OSTRC-H.JP including both the original version of question 2 and updated version of question 2 every Sunday for 10 consecutive weeks to assess the consequences of the change in wording [12]. The questionnaire was set up on the Google form platform and the web link was distributed to the athletes via email.

### Changes to questionnaire logic and answer categories

By applying gatekeeper logic, it was reported that the number of health problems identified was reduced in the Norwegian version of the questionnaire [12]. This is because it eliminates the opportunity for athletes to report ‘full participation without health problems’ in question 1 and then inconsistently report the existence of a health problem in subsequent questions [12].

To assess the consequences of changes to the questionnaire logic in Japanese questionnaires, we calculated the number of health problems identified when gatekeeper logic was and was not applied, using 981 OSTRC-H.JP responses from Japanese athletes in this cohort study. In addition, 604 OSTRC-H.JP responses from Japanese athletes to assess the validity and reliability of the questionnaire in an earlier study [18] were also included in the analyses.

### Statistical analysis

Basic information of the participants were presented in mean and standard deviation. The response rate was presented in percentages and 95% confidence interval (95%CI) for all athletes, irrespectively of the sport. The prevalence of health problems was calculated once a week by dividing the number of athletes reporting any type of problem by the number of questionnaire respondents [7, 13]. The prevalence of substantial health problems was also calculated using these measures, with substantial health problems defined as those leading to moderate or severe reductions in training volume, or moderate or severe reductions in sports performance, or complete inability to participate in sport [7, 13].

Cohen’s kappa using equal weights was calculated to analyse the level of agreement between the original version of question 2 and updated version of question 2 with more substantial changes [12]. The kappa coefficients were defined as follows: 0.00 to 0.20 = slight agreement, 0.21 to 0.40 = fair agreement, 0.41 to 0.60 = moderate agreement, 0.61 to 0.80 = substantial agreement, and 0.81 to 1.00 = almost perfect agreement [22]. To assess the consequences of applying gatekeeper logic, we calculated the number of health problems and the differences as a percentage [12]. Statistical analysis was performed using SPSS version 26.0 (IBM Corporation, Armonk, NY, USA) with the significant level set at *p*<0.05.

## Results

### More substantial changes

To assess the consequences of the change in question 2 wording, 101 participants were followed over 10 weeks. The average response rate to the OSTRC-H.JP during the 10 weeks was 97.1% (95%CI, 96.1–98.1). The average prevalence of health problems was 32.6% (95%CI, 29.7–35.6) and substantial health problems which caused moderate/severe reductions in training volume or sports performance, or complete inability to participate in training or competition was 13.9% (95%CI, 11.7–16.0).

During the 10 weeks, a total of 981 questionnaire responses were collected, of which 342 included a health problem. The kappa coefficient for comparison between the original version of question 2 and the updated version of question 2 was 0.84. The distribution between the responses to the original and updated versions of question 2 is shown in Table 2.

**Table 2:** Distribution between the responses to the original and updated versions of question 2 in Japanese questionnaire.

		Updated version: Training or competition modification				
		2a	2b	2c	2d	2e*
Original version: Training reduction	2a	196	4	1	0	0
	2b	9	27	2	2	0
	2c	1	1	13	5	1
	2d	0	0	0	24	4
	2e	0	1	0	2	49

Column and row headings 2a–e represent response categories to question 2 as shown in S1 and S2 Files.

*A fifth response category (could not participate at all) to the updated version was included in this analysis.

### Changes to questionnaire logic and answer categories

A total of 1585 questionnaire responses were used to assess the consequences of changes to the questionnaire logic, of which 981 were from this cohort study to assess the consequences of the change in question 2 and 604 from an earlier study [18]. From these responses, 568 health problems were identified, including 545 injuries and 23 illnesses.

For all health problems, 5.0% of the total number of injury cases and 8.7% of illness cases were missed when gatekeeper logic was used. Among substantial health problems, there was a 1.7% decrease in the number of cases of injury but no decrease in the number of cases of illness. For time loss health problems, where respondents were unable to train or compete for more than one day in the previous seven days due to that health problem identifying an additional question, injuries showed a 2.7% decrease and illnesses showed a 7.1% decrease in the number of cases. The comparison of the number of injuries and illnesses identified when gatekeeper logic was and was not applied to question 1 is described in Table 3.

**Table 3:** Comparison of the number of health problems identified with and without the application of gatekeeper logic to question 1 in Japanese questionnaire.

	No logic	Gatekeeper logic	Difference (%)
All health problems			
Injuries	545	518	5.0
Illnesses	23	21	8.7
Substantial health problems			
Injuries	240	236	1.7
Illnesses	13	13	0.0
Time loss health problems			
Injuries	187	182	2.7
Illnesses	14	13	7.1

## Discussion

In this study, the Japanese versions of the OSTRC questionnaires were updated and the impact of more substantial changes and changes to questionnaire logic were analysed with Japanese athletes. The more substantial changes to the wording of question 2 were assessed over a 10-week cohort study. The kappa coefficient, which measures the level of agreement between responses to question 2 of the original and updated versions, was high. By applying gatekeeper logic, there was a remarkable reduction in the number of injuries and illnesses of all health problems in the OSTRC-H.JP but less reduction in substantial health problems and time loss health problems.

Throughout the 10-week cohort study, 342 health problems were identified from the responses. The kappa coefficient, used to assess the level of agreement between the original and updated versions of question 2, was over 0.81, showing almost perfect agreement [22]. Question 2 of the original version asked to what extent athletes reduced training volume due to health problems [7, 13]. However, reducing training volume is only one way for an athlete to modify their normal sports participation in addressing a health problem, and other modifications such as reducing intensity or changing the type of training may have been missed [12]. To address this, a change to the wording in question 2 was effected. In a study conducted on three Dutch National Olympic programmes to assess the level of agreement between responses to question 2, Clarsen *et al*. [12] reported that the kappa coefficient was 0.55. The aforementioned researchers showed that the main inconsistencies between the original and updated versions occurred with the least severe health problems, which had little or no influence on training [12]. The results of this study showed a higher kappa coefficient than that of Clarsen *et al*. [12], but there was a similar tendency for inconsistencies to occur in the least severe health problems.

By applying gatekeeper logic, there was a 5.0% reduction in the number of injuries and a 8.7% reduction in the number of illnesses among all health problems. However, there was less of a decrease among substantial health problems and time loss health problems: approximately 1.6% (1.7% in injuries and 0% in illnesses) and 3.0% (2.7% in injuries and 7.1% in illnesses), respectively. Clarsen *et al*. [12] described a problem when respondents were required to answer all four key questions, as their responses were not always consistent. By applying this logic, discrepancies in the responses to the four key questions as well as unnecessary responder burden can be reduced by guaranteeing that athletes only answer questions relevant to their current health conditions [12]. A study comparing the number of injuries and illnesses with and without gatekeeper logic based on 13888 OSTRC-H responses from elite Norwegian athletes reported an approximately 13% reduction in the number of health problems (12.0% in injuries and 15.2% in illnesses) with gatekeeper logic application in all health problems [12]. It is, however, also indicated that the missing cases were mostly of minor severity, as 98.5% of substantial health problems were reported using gatekeeper logic [12]. In the present study, there was also a remarkable decrease in the number of cases of minor severity health problems, because the decrease was less with substantial and time loss health problems. Therefore, the use of gatekeeper logic in the Japanese versions of the questionnaires can reduce the discrepancies in responses to minor health problems.

In the current study, the Japanese versions of the questionnaires were updated successfully, although it has some limitations. First, 981 responses from the cohort study and 604 responses from an earlier study [18] were analysed to assess the impact of the changes to questionnaire logic for OSTRC-H.JP. This was far lesser than the number of responses obtained in a similar previous study involving Norwegian athletes [12]. Although the reduction in the number of health problems when the gatekeeper logic was applied showed the same tendency in both cases, the percentages shown by the reduction in one health problem were larger in this study because of the small number of responses analysed. Additionally, all the responses obtained from the present cohort study were from basketball and football players, whereas 604 responses in an earlier study were basketball, football, handball, gymnastics, and kendo athletes [18]. Although the questionnaires are intended for use in a variety of sports, the responses from only few sports were analysed in this study.

## Conclusion

In this study, the Japanese versions of the OSTRC questionnaires were updated and minor and more substantial changes were implemented. Changes to the questionnaire logic helped to reduce the discrepancies in responses to minor health problems. These changes will make it easier for Japanese athletes to complete the questionnaires and improve the quality of collected data.

## Supporting information

S1 File The original and updated versions of the Japanese questionnaire for registration of overuse injuries. (PDF)

S2 File The original and updated versions of the Japanese questionnaire for registration of health problems. (PDF)

S1 Data Data set for more substantial changes. (XLSX)

S2 Data Data set for changes to questionnaire logic. (XLSX)

## References

[1] van Mechelen W, Hlobil H, Kemper HC. Incidence, severity, aetiology and prevention of sports injuries. A review of concepts. Sports Med. 1992; 14(2): 82–99. 10.2165/00007256-199214020-00002 1509229

[2] Bahr R, Clarsen B, Derman W, Dvorak J, Emery CA, Finch CF, et al. International Olympic Committee consensus statement: methods for recording and reporting of epidemiological data on injury and illness in sport 2020 (including STROBE Extension for Sport Injury and Illness Surveillance (STROBE-SIIS)). Br J Sports Med. 2020; 54(7): 372–389. 10.1136/bjsports-2019-101969 32071062 PMC7146946

[3] Fuller CW, Ekstrand J, Junge A, Andersen TE, Bahr R, Dvorak J, et al. Consensus statement on injury definitions and data collection procedures in studies of football (soccer) injuries. Br J Sports Med. 2006; 40(3): 193–201. 10.1136/bjsm.2005.025270 16505073 PMC2491990

[4] Fuller CW, Molloy MG, Bagate C, Bahr R, Brooks JHM, Donson H, et al. Consensus statement on injury definitions and data collection procedures for studies of injuries in rugby union. Br J Sports Med. 2007; 41(5): 328–331. 10.1136/bjsm.2006.033282 17452684 PMC2659070

[5] Murray A, Junge A, Robinson PG, Bizzini M, Bossert A, Clarsen B, et al. International consensus statement: methods for recording and reporting of epidemiological data on injuries and illnesses in golf. Br J Sports Med. 2020; 54(19): 1136–1141. 10.1136/bjsports-2020-102380 32847810 PMC7513248

[6] Junge A, Engebretsen L, Alonso JM, Renström P, Mountjoy M, Aubry M, et al. Injury surveillance in multi-sport events: the International Olympic Committee approach. Br J Sports Med. 2008; 42(6): 413–421. 10.1136/bjsm.2008.046631 18390916

[7] Clarsen B, Myklebust G, Bahr R. Development and validation of a new method for the registration of overuse injuries in sports injury epidemiology: the Oslo Sports Trauma Research Centre (OSTRC) Overuse Injury Questionnaire. Br J Sports Med. 2013; 47(8): 495–502. 10.1136/bjsports-2012-091524 23038786

[8] Olsen OE, Myklebust G, Engebretsen L, Bahr R. Injury pattern in youth team handball: a comparison of two prospective registration methods. Scand J Med Sci Sports. 2006; 16(6): 426–432. 10.1111/j.1600-0838.2005.00484.x 17121645

[9] Bjørneboe J, Bahr R, Andersen TE. Gradual increase in the risk of match injury in Norwegian male professional football: A 6-year prospective study. Scand J Med Sci Sport. 2014; 24(1): 189–196. 10.1111/j.1600-0838.2012.01476.x 22582981

[10] Stubbe JH, Van Beijsterveldt AMMC, Van Der Knaap S, Stege J, Verhagen EA, Van Mechelen W, et al. Injuries in professional male soccer players in the Netherlands: A prospective cohort study. J Athl Train. 2015; 50(2): 211–216. 10.4085/1062-6050-49.3.64 25531144 PMC4495432

[11] Bahr R. No injuries, but plenty of pain? On the methodology for recording overuse symptoms in sports. Br J Sports Med. 2009; 43(13): 966–972. 10.1136/bjsm.2009.066936 19945978

[12] Clarsen B, Bahr R, Myklebust G, Andersson SH, Docking SI, Drew M, et al. Improved reporting of overuse injuries and health problems in sport: an update of the Oslo Sport Trauma Research Center questionnaires. Br J Sports Med. 2020; 54(7): 390–396. 10.1136/bjsports-2019-101337 32060142

[13] Clarsen B, Rønsen O, Myklebust G, Flørenes TW, Bahr R. The Oslo sports trauma research center questionnaire on health problems: A new approach to prospective monitoring of illness and injury in elite athletes. Br J Sports Med. 2014; 48(9): 754–760. 10.1136/bjsports-2012-092087 23429267

[14] Ekman E, Frohm A, Ek P, Hagberg J, Wirén C, Heijne A. Swedish translation and validation of a web-based questionnaire for registration of overuse problems. Scand J Med Sci Sport. 2015; 25(1): 104–109. 10.1111/sms.12157 24313387

[15] Jorgensen JE, Rathleff CR, Rathleff MS, Andreasen J. Danish translation and validation of the Oslo Sports Trauma Research Centre questionnaires on overuse injuries and health problems. Scand J Med Sci Sport. 2016; 26(12): 1391–1397. 10.1111/sms.12590 26631937

[16] Hirschmüller A, Steffen K, Fassbender K, Clarsen B, Leonhard R, Konstantinidis L, et al. German translation and content validation of the OSTRC Questionnaire on overuse injuries and health problems. Br J Sports Med. 2017; 51(4): 260–263. 10.1136/bjsports-2016-096669 27797733

[17] Nagano Y, Kobayashi-Yamakawa K, Higashihara A, Yako-Suketomo H. Japanese translation and modification of the Oslo Sports Trauma Research Centre overuse injury questionnaire to evaluate overuse injuries in female college swimmers. PLoS One. 2019; 14(4): e0215352. 10.1371/journal.pone.0215352 30986226 PMC6464216

[18] Mashimo S, Yoshida N, Hogan T, Takegami A, Hirono J, Matsuki Y, et al. Japanese translation and validation of web-based questionnaires on overuse injuries and health problems. PLoS One. 2020; 15(12): e0242993. 10.1371/journal.pone.0242993 33270675 PMC7714361

[19] Beaton DE, Bombardier C, Guillemin F, Ferraz MB. Guidelines for the process of cross-cultural adaptation of self-report measures. Spine. 2000; 25(24): 3186–3191. 10.1097/00007632-200012150-00014 11124735

[20] Beaton DE, Bombardier C, Guillemin F, Ferraz MB. Recommendations for the cross-cultural adaptation of the DASH & QuickDASH outcome measures. Institute for Work and Health; 2007.

[21] Wild D, Grove A, Martin M, Eremenco S, McElroy S, Verjee-Lorenz A, et al. Principles of good practice for the translation and cultural adaptation process for patient-reported outcomes (PRO) measures: report of the ISPOR task force for translation and cultural adaptation. Value Heal. 2005; 8(2): 94–104. 10.1111/j.1524-4733.2005.04054.x 15804318

[22] Landis JR, Koch GG. The measurement of observer agreement for categorical data. Biometrics. 1977; 33(1): 159–174. 843571

