# Supplementary material for: An update of the Japanese Oslo Sports Trauma Research Center questionnaires on overuse injuries and health problems
Source: PLoS One. 2021 Apr 1;16(4):e0249685. doi: 10.1371/journal.pone.0249685 (PMC8016239; doi:10.1371/journal.pone.0249685)
Supplement: S1 File — (PDF) [file pone.0249685.s001.pdf]

障害（オーバーユース）に関する質問紙

原版 (Original version: OSTRC-O.JP)*	改訂版 (Updated version: OSTRC-O2.JP)
あなたの膝に問題があるかどうかに関わらず全ての質問に答えてください。選択肢の中から最適なものを選び、わからない場合でも最もあてはまる答えを選ぶように努めてください。<br>「膝の問題」とは、片方あるいは両方の膝に関する痛み、うずき、硬さ、腫れ、不安定感/膝崩れ、ロッキング（引っかかり）、またはその他の症状のこととします。	あなたの[部位を挿入、例えば膝など]に問題があるかどうかに関わらず全ての質問に答えてください。選択肢の中から最適なものを選び、わからない場合でも最もあてはまる答えを選ぶように努めてください。<br>「[部位]の問題」とは、[部位]に関する[主な症状や傷害の影響を挿入、例えば痛み、うずき、硬さ、腫れ、不安定感/膝崩れ、ロッキングなど]、またはその他の症状のこととします。
質問１<br>過去 1 週間に膝の問題により、通常の練習や試合への参加に影響が出ましたか？<br><br>a. 膝の問題はなく、全ての練習や試合に参加することができた。<br>b. 膝の問題はあったが、全ての練習や試合に参加することができた。<br>c. 膝の問題があり、練習や試合への参加を減らした。<br>d. 膝の問題があり、練習や試合を行うことができなかった。	質問１—参加<br>過去 7 日間に[部位]の問題により、練習や試合への参加に影響が出ましたか？<br><br>a. [部位]の問題はなく、全ての練習や試合に参加することができた。<br>b. [部位]の問題はあったが、全ての練習や試合に参加することができた。<br>c. [部位]の問題があり、練習や試合への参加を減らした。<br>d. [部位]の問題があり、練習や試合を行うことができなかった。
質問２<br>過去 1 週間に膝の問題により、どの程度練習量を減らしましたか？<br><br>a. 全く減らさなかった。<br>b. 少し減らした。<br>c. 半分程度減らした。<br>d. かなり減らした。<br>e. 練習や試合が全くできなかった。	質問２—練習/試合の変更<br>過去 7 日間に[部位]の問題により、どの程度練習や試合を変更しましたか？<br><br>a. 全く変更しなかった。<br>b. 少し変更した。<br>c. 半分程度変更した。<br>d. かなり変更した。
質問３<br>過去 1 週間に膝の問題が、どの程度パフォーマンスに影響しましたか？<br><br>a. 全く影響しなかった。<br>b. 少し影響した。<br>c. ある程度影響した。<br>d. かなり影響した。<br>e. 全く練習や試合ができない程、影響した。	質問３—パフォーマンス<br>過去 7 日間に[部位]の問題が、どの程度パフォーマンスに影響しましたか？<br><br>a. 全く影響しなかった。<br>b. 少し影響した。<br>c. ある程度影響した。<br>d. かなり影響した。
質問４<br>過去 1 週間に経験した膝の痛みはどの程度でしたか？<br><br>a. 全く痛みはなかった。<br>b. 少し痛みがあった。<br>c. 中程度の痛みがあった。<br>d. かなりの痛みがあった。	質問４—痛み<br>過去 7 日間に経験した[部位]の痛みはどの程度でしたか？<br><br>a. 全く痛みはなかった。<br>b. 少し痛みがあった。<br>c. 中程度の痛みがあった。<br>d. かなりの痛みがあった。

*Mashimo et al. [18]より引用.
質問紙の変更箇所は赤字で示している.
