# Supplementary material for: An update of the Japanese Oslo Sports Trauma Research Center questionnaires on overuse injuries and health problems
Source: PLoS One. 2021 Apr 1;16(4):e0249685. doi: 10.1371/journal.pone.0249685 (PMC8016239; doi:10.1371/journal.pone.0249685)
Supplement: S2 File — (PDF) [file pone.0249685.s002.pdf]

身体上の問題に関する質問紙

原版 (Original version: OSTRC-H.JP)*	改訂版 (Updated version: OSTRC-H2.JP) †
過去１週間で身体上の問題があったかどうかに関わらず全ての質問に答えてください。選択肢の中から最適なものを選び、わからない場合でも最もあてはまる答えを選ぶように努めてください。<br>もし複数の疾病あるいは傷害がある場合には、過去１週間で最も悪かった問題について答えてください。その他の問題については質問紙の最後に回答する機会があります。	過去７日間で身体上の問題があったかどうかに関わらず全ての質問に答えてください。選択肢の中から最適なものを選び、わからない場合でも最もあてはまる答えを選ぶように努めてください。<br>身体上の問題とは、スポーツ参加やパフォーマンスへの影響、または医学的な処置を必要としたかどうかに関係なく、通常の健康状態よりも低下したとみなされるあらゆる状態のことです。これには、傷害、疾病、痛み、または精神的な健康状態が含まれますが、これらに限定されません。<br>もし複数の身体上の問題がある場合には、過去７日間で最も悪かった問題から答えてください。その他の問題については質問紙の最後に回答する機会があります。
質問１<br>過去１週間に傷害、疾病、あるいはその他の身体上の問題により、通常の練習や試合への参加に影響が出ましたか？<br><br>a. 身体上の問題はなく、全ての練習や試合に参加することができた。<br>b. 傷害/疾病はあったが、全ての練習や試合に参加することができた。<br>c. 傷害/疾病があり、練習や試合への参加を減らした。<br>d. 傷害/疾病があり、練習や試合を行うことができなかった。	質問１—参加<br>過去７日間に傷害、疾病、あるいはその他の身体上の問題により、練習や試合への参加に影響が出ましたか？<br><br>a. 身体上の問題はなく、全ての練習や試合に参加することができた。<br>b. 身体上の問題はあったが、全ての練習や試合に参加することができた。<br>c. 身体上の問題があり、練習や試合への参加を減らした。<br>d. 身体上の問題があり、練習や試合を行うことができなかった。
質問２<br>過去１週間に傷害、疾病、あるいはその他の身体上の問題により、どの程度練習量を減らしましたか？<br><br>a. 全く減らさなかった。<br>b. 少し減らした。<br>c. 半分程度減らした。<br>d. かなり減らした。<br>e. 練習や試合が全くできなかった。	質問２—練習/試合の変更<br>過去７日間に傷害、疾病、あるいはその他の身体上の問題により、どの程度練習や試合を変更しましたか？<br><br>a. 全く変更しなかった。<br>b. 少し変更した。<br>c. 半分程度変更した。<br>d. かなり変更した。
質問３<br>過去１週間に傷害、疾病、あるいはその他の身体上の問題が、どの程度パフォーマンスに影響しましたか？<br><br>a. 全く影響しなかった。<br>b. 少し影響した。<br>c. ある程度影響した。<br>d. かなり影響した。<br>e. 全く練習や試合ができない程、影響した。	質問３—パフォーマンス<br>過去７日間に傷害、疾病、あるいはその他の身体上の問題が、どの程度パフォーマンスに影響しましたか？<br><br>a. 全く影響しなかった。<br>b. 少し影響した。<br>c. ある程度影響した。<br>d. かなり影響した。
質問４<br>過去 １ 週間に経験した身体上の問題の症状はどの程度でしたか？<br><br>a. 全く症状はなかった。<br>b. 少し症状があった。<br>c. 中程度の症状があった。<br>d. かなりの症状があった。	質問４—症状<br>過去７日間に経験した身体上の問題の症状はどの程度でしたか？<br><br>a. 全く症状はなかった。<br>b. 少し症状があった。<br>c. 中程度の症状があった。<br>d. かなりの症状があった。

*Mashimo et al. [18]より引用.

†改訂版では，質問４以降に研究や臨床の必要性に応じて追加質問を設定することが可能である.

質問紙の変更箇所は赤字で示している.
